# Role of Echocardiography Before Transcatheter Aortic Valve Implantation (TAVI)

**DOI:** 10.1007/s11886-016-0715-z

**Published:** 2016-03-09

**Authors:** Sveeta Badiani, Sanjeev Bhattacharyya, Guy Lloyd

**Affiliations:** Barts Heart Centre, St Bartholomew’s Hospital, West Smithfield, London, EC1A 7BE UK; Institute for Cardiovascular Sciences, University College London, Gower Street, London, WC1E 6BT UK; Institute for Advanced Imaging, Queen Mary University of London, Mile End Road, London, E1 4NS UK

**Keywords:** Aortic stenosis, Transcatheter aortic valve implantation, Echocardiography

## Abstract

Aortic stenosis (AS) is the most common primary valve disorder in the elderly with an increasing prevalence; transcatheter aortic valve implantation (TAVI) has become an accepted alternative to surgical aortic valve replacement (AVR) in the high risk or inoperable patient. Appropriate selection of patients for TAVI is crucial and requires a multidisciplinary approach including cardiothoracic surgeons, interventional cardiologists, anaesthetists, imaging experts and specialist nurses. Multimodality imaging including echocardiography, CT and MRI plays a pivotal role in the selection and planning process; however, echocardiography remains the primary imaging modality used for patient selection, intra-procedural guidance, post-procedural assessment and long-term follow-up. The contribution that contemporary transthoracic and transoesophageal echocardiography make to the selection and planning of TAVI is described in this article.

## Introduction

Aortic stenosis is the commonest left-sided valve lesion [1] and affects 12.4 % of patients over the age of 75 [2]. Severe aortic stenosis is present in 3.4 % of patients [2]. The most frequent aetiology is calcification of a normal tri-leaflet valve or secondary to a congenital bicuspid valve [3–5], which has a prevalence of 1 % [6]. Rheumatic aortic stenosis is less prevalent in the developed world, although it remains a common cause of mortality in more economically challenged regions. The prognosis in patients with symptomatic aortic stenosis is poor, with the interval from the onset of symptoms to the time of death being approximately 2 years in individuals with heart failure, 3 years in those with syncope and 5 years in those with angina [7].

Severe aortic stenosis is conventionally defined as a peak aortic velocity >4 m/s, a mean gradient >40 mmHg and a valve area <1 cm^2^ [8••]. Class I indications for aortic valve replacement are symptomatic severe aortic stenosis, asymptomatic patients with severe aortic stenosis and left ventricular ejection fraction <50 % and patients with severe aortic stenosis undergoing cardiac surgery for other indications [8••].

The majority of aortic valve replacements are surgical aortic valve replacement (AVR). However, in high-risk patient groups, factors such as increasing age, prior cardiac surgery and other co-morbidities, such as heart failure, respiratory and renal diseases, are associated with a high potential for operative mortality and morbidity [9, 10]. AVR has historically been less frequently performed in these patients [11], and hence, one third of patients with symptomatic aortic stenosis have in the past been denied surgical intervention [12]. Transcatheter aortic valve implantation (TAVI) was first performed in 2002 as a less invasive approach [13] and is now recommended as an alternative strategy for patients in high-risk surgical groups [14]. This practice is supported by clear randomised trial evidence demonstrating superiority over medical treatment and equivalence to conventional surgery in high-risk subgroups [7, 15, 16•, 17]. The ongoing Placement of AoRTic TraNscathetER Valve (PARTNER) II trial will assess an intermediate risk cohort comparing TAVI versus surgical AVR in a randomised 1:1 fashion [18].

TAVI was initially performed almost exclusively under general anaesthesia, and transoesphageal echocardiography was an integral guide for the pre-operative procedure. Recent years have seen an increasing trend for performing the procedure under conscious sedation, and the role of echo has evolved, so now, it plays a major role in case selection and procedure planning, with an increasing emphasis on transthoracic echocardiography (TTE).

## Echocardiography Guidance to Case Selection for TAVI

- Severity of aortic stenosis

Aortic stenosis is a disease both of the valve and the ventricular response to the chronic afterload circumstance. The classical definition of severe aortic stenosis is still reflected in the current guidelines, that is a *V*_max_ >4 m/s, mean gradient >40 mmHg and AVA <1 cm^2^, in the presence of preserved left ventricular function [8••]. When indexed for body surface area, a valve area of 0.6 cm^2^/m^2^ is also considered the threshold for severe aortic stenosis (AS). This is conventionally described as high-gradient aortic stenosis (HGAS). However, the myocardial response may develop in a way that results in reduced flow through the valve; this results in a significantly lower transvalvular gradient into the ‘moderate’ or even ‘mild’ range. Situations where the aortic valve gradients suggest a lesser severity of aortic stenosis than the valve area are very common [19], and this may be due to a low stroke volume in patients with either a dilated left ventricle and reduced ejection fraction (low-flow, low-gradient AS (LFLGAS)), or where there is excessive hypertrophy leading to a small ventricular cavity size with normal ejection fraction [20], but reduced stroke volume (paradoxical low-flow aortic stenosis (PLFLGAS)).

These low-gradient patients may have truly severe AS; alternatively, the reduction of flow may be the cause of the low valve area, so-called *pseudo severe AS*. This is a critical distinction because in true severe AS, intervention on the aortic valve is likely to result in clinical improvement, whereas if the problem primarily lies at the level of the myocardium, this is less likely.

Sorting these discrepant results out requires the integration of echocardiography, other imaging modalities and good clinical sense. Evaluation starts with a visual assessment of the structure, calcification and mobility of the aortic valve. A relatively mobile valve is unlikely to represent severe aortic stenosis no matter what the Doppler calculations demonstrate. In patients with impaired ejection fraction, increasing flow through the valve using dobutamine stress echo (DSE) is well established and has been recommended as a class IIa indication in managing patients with valvular heart disease [8••, 14]. If a 20 % increase in stroke volume is associated with the development of a gradient of 40 mmHg or a peak velocity of greater than 4 m/s, with an unchanging valve area, then, the AS is severe and intervention is warranted.

In PLFLGAS, the situation is more complex and measurement errors are more frequent. Even when measurements are made correctly, the anatomy of the outflow tract may cause significant variability. The anteroposterior dimension of the left ventricular outflow tract (LVOT) used in calculating the aortic valve area using the continuity equation is susceptible to measurement error, and the effect of this is amplified as the measurement is squared to describe the LVOT area [21]. Not only that, the LVOT is rarely circular with the aortic prosthesis (AP) dimension often being the minor dimension [22]. Confirming normal flow through the valve (>35 mls/m^2^) makes significant AS unlikely, but where the clinical situation remains unclear, either DSE or cardiac CT to evaluate the calcium score of the aortic valve is helpful [23].

Whilst there is clear evidence of benefit from TAVI in patients with HGAS, TAVI may be an attractive option in the patients with low-gradient, low ejection fraction aortic stenosis, compared with surgical AVR [24]. The procedure has been associated with enhanced recovery in patients with reduced ejection fraction [25]. There is limited data as to whether patients with paradoxical low-flow aortic stenosis will benefit from TAVI [26, 27, 28•]. A study by Sullivan et al. showed that symptomatic patients with paradoxical low-flow aortic stenosis demonstrated a functional improvement at 1-year post-TAVI. Patients with low ejection fraction and low-flow AS also had functional improvement 1-year post-TAVI, although the left ventricular (LV) function improvement, although significant, was less than that observed with high-gradient aortic stenosis [29].

Although post-procedure outcome is generally worse in the low-gradient, low-flow groups, survival is still improved with TAVI compared to medical management and is similar with TAVI and surgical AVR, in patients with paradoxical low-flow aortic stenosis. TAVI reduced 1-year mortality from 66 to 35 %, in these patients [26]. Data from the German TAVI registry found that in high-risk patients with low-gradient severe AS, TAVI associated with a significantly higher mortality at 30 days and at 1 year, although long-term survivors did benefit from functional improvement and improved quality of life [29]. Low-flow, low-gradient aortic stenosis has a strong impact on 6-month and 1-year mortality; however, there are considerable haemodynamic and clinical improvements. Therefore, it is important to weigh up the risks and benefits of TAVI in every patient with low-flow aortic stenosis [30].

(b) Aortic valve cusp anatomy

Imaging assessment of the number and arrangement of cusps prior to TAVI is essential. The asymmetry of the annular ring of a bicuspid valve can result in an elliptical annular shape with associated eccentric calcium distribution [31]. The TAVI prosthesis may, therefore, not expand fully, leading to prosthesis misplacement and paravalvular regurgitation [32]. Dilatation of the ascending aorta is also common in patients with bicuspid aortic valves [33], and TAVI may therefore be associated with an increased risk of aortic dissection [34]. The PARTNER trial excluded all patients with bicuspid aortic valves, as there was concern that the valve may distort the prosthesis, leading to significant paravalvular regurgitation [7]. There have, however, been multiple descriptions of successful TAVI in bicuspid severe AS [35].

A study carried out by Wijesinghe in a cohort of 11 patients with bicuspid severe aortic stenosis undergoing TAVI showed an improvement in valve area and mean gradient. Two patients were found to have moderate paravalvular aortic regurgitation and one patient required surgery for valve migration, over a 30-day follow-up period [36]. Phan et al. carried out a systematic review and meta-analysis on 149 patients undergoing TAVI. They found no difference in 30-day mortality, post-TAVI gradients, moderate to severe paravalvular AR, bleeding or vascular complications in patients with bicuspid valves, suggesting that TAVI is feasible in these patients [37•].

The results of the German TAVI registry showed that there was an increased rate of paravalvular aortic regurgitation in patients with bicuspid compared to tricuspid aortic valves (25 vs 15 %, *p* = 0.05), although the 30-day or 1-year mortality was not elevated [38]. The Poland National Registry investigators found that the post-procedure mean transvalvular gradient, severity of aortic regurgitation and 30-day and 1-year mortality were similar between patients with bicuspid and tricuspid valves [39].

These results have encouraged an increasing number of operators to undertake TAVI more freely in this group of patients, and the presence of a bicuspid valve should be considered a risk marker for complications and suboptimal deployment rather than an absolute contraindication. Diagnosis of bicuspid anatomy with 2D echocardiography is often challenging, and 3D techniques are often needed to assess the elliptical geometry of the annulus and for accurate dimension measurement. Other factors in device selection include the presence or absence of a concomitant aortopathy and the location and dominance of the coronary arteries, which are more likely to have anatomic variations with bicuspid aortic valve disease [40].

(c) Mitral regurgitation

Patients with severe aortic stenosis may also have significant mitral regurgitation (MR) [41], which is typically left untreated in the TAVI population. There are multiple causes of mitral regurgitation, and its mechanisms are due to an organic (due to a structurally abnormal valve) or functional cause [42]. Patients with severe aortic stenosis have a high prevalence of coronary artery disease, which may result in ischaemic mitral regurgitation. End-stage aortic stenosis may also lead to left ventricular dilatation, and these factors, along with concomitant aortic regurgitation, can influence the presence and severity of functional MR [43].

Moderate to severe MR occurs in approximately 20 % of patients undergoing TAVI, and its severity improves in around 50 % of patients after the intervention, especially in those with non-structural MR associated with adaptive changes in LV geometry [44].

Studies of patients with severe MR suggested an increase in early mortality after TAVI [43, 44], due to poor post-procedure haemodynamics and heart failure, whilst other studies that included patients with moderate or severe MR as part of the significant MR group failed to show this association [45, 46]. With regard to late mortality, patients with moderate or severe MR had similar mortality rates compared to those with no or mild MR [45]. In contrast to these results, with increasing grades of MR severity, there was an increasing risk of 1-year mortality from the German and Italian TAVI registries [47, 48].

(d) Pulmonary arterial hypertension and right ventricular dysfunction

Pulmonary hypertension may be present in patients with severe AS, due to transmission of increased left ventricular diastolic or left atrial pressures, associated mitral regurgitation or an increase in pulmonary vascular tone, which can result in right heart failure. It is an accepted predictor of poor outcome after surgical AVR, and there is growing evidence that pulmonary hypertension and tricuspid regurgitation have an adverse impact on prognosis in patients undergoing TAVI [49]. A recent large study of 155 patients, however, showed that pre-procedural right ventricular dysfunction did not adversely influence medium-term outcome following TAVI [50].

(e) Valve in valve TAVI

Redo surgery is the standard treatment for degenerative aortic bioprostheses. This, however, carries an operative mortality risk of 1.5–23 %, depending on patient age, number of previous operations, left ventricular dysfunction and technical difficulties caused by adhesions [51]. TAVI may be an attractive option in these high-risk surgical patients, and indications for valve in valve TAVI include bioprosthetic stenosis, regurgitation or both.

Previous studies have confirmed the feasibility of valve in valve TAVI for patients with failing bioprostheses [52–54]. A registry of 202 patients undergoing valve in valve TAVI showed that 84.1 % of patients reported an improvement in symptoms and were NYHA functional class I/II early after the procedure. However, a high rate of device malposition, and elevated post-procedural gradients, was observed. The relatively high malposition rate may be secondary to the relative lack of valvular calcification and, in some cases, difficulty in defining the optimal target for implantation during the procedure, particularly in stentless bioprostheses, in which no anatomic markers are available [55].

Similar to the TAVI workup in patients with native AS, patients being considered for valve in valve TAVI should undergo imaging with TTE ± transoesophageal (TOE) and CT. Aortic root anatomy, coronary ostial position and the presence of pannus and leaflet calcification should be identified [56]. Transcatheter heart valves are usually oversized to achieve stability, prevent migration of the valve and to minimise paravalvular regurgitation.

## TAVI Procedure Planning

Once an assessment of the severity of aortic stenosis incorporating both valvular and other echocardiographic factors has been performed, echo has an important role alongside other imaging modalities, especially cardiac CT, in procedure planning. A multidisciplinary approach and the use of multiple imaging modalities to fully delineate the anatomy of the aortic valve, aorta and peripheral vasculature is essential. Echocardiography has also played a major role intra-procedurally and in follow-up [57]. This role has recently been disputed by trial data that fails to show an outcome benefit nor reduced risk associated with TOE guidance. Transoesophageal echo measurement, as well as pre-procedure cardiac CT, may have limitations in precise annular sizing; however, TOE may be helpful in select cases. Balloon valvuloplasty during the procedure may also be used to assist in annular sizing and in device selection [58].

- Distribution of calcium

Echocardiography is useful in demonstrating the presence and distribution of calcium. Aortic valve calcification increases the risk of gaps between the prosthesis and the native valve, which can lead to paravalvular regurgitation, although a degree of annular calcification is present in all patients with degenerative aortic stenosis [59]. A large amount of calcification at the edge of the native valvular leaflets may increase the risk of coronary occlusion by displacement over the coronary ostium. Furthermore, heavy calcification in the sinotubular junction may cause restriction during balloon expansion at the aortic end and consequently affects ventricular displacement of the device at the time of deployment [60, 61]. A higher incidence of post-procedure aortic regurgitation is seen with significant annular and commissural calcification [62]. Extensive calcification especially in the subvalvular position is one of the major predictors of annulus or sinus rupture during implantation. The mechanisms are unclear and the subvalvular areas appear to predict a higher risk, perhaps because valvular calcification can be accommodated within the sinus of valsalva. Localised calcification at the valve tips is a well recognised although rare cause of aortic rupture and contained haematoma.

(b) Annulus size measurement

The aortic annulus is described as a virtual ring formed by joining the basal attachments of the aortic cusps [63]. Accurate understanding of the anatomy of the aortic annulus and measurement of the annular dimension is key as it determines eligibility for TAVI and guides the type and size of the valve to be used. Implantation of an appropriately sized prosthesis will reduce the frequency of serious complications [64]. Underestimation of annular size can lead to selection and deployment of a valve that is too small which can lead to prosthesis migration and paravalvular regurgitation [65]. Overestimation can lead to incomplete deployment, valvular and paravalvular regurgitation or annular rupture, which has been described in around 5 % of patients undergoing TAVI with a balloon-expandable valve [66]. The objective is to deploy a valve of sufficient size to reduce paraprosthetic regurgitation but not exceeding >20 % greater than the measured annulus, where ruptures and contained ruptures become much more common.

The annular AP diameter has traditionally been measured in the long-axis view in systole at the hinge points of the leaflets into the LVOT with a trailing edge to leading edge convention. During TOE, a long-axis view (120–140°) is used to measure the annular diameter in systole at the level of the basal attachment of the valve cusps. 2D methods, however, have their limitations in sizing, as a circular rather than an elliptical annulus is assumed [61]. A 3D TOE study showed that the LVOT is oval in shape in 90 % of patients [67]. There is good reproducibility with all echocardiographic modalities in aortic annulus measurement, the highest being with 3D TOE. Measurements made by 3D techniques alter the choice of prosthesis size and have proven superior for the prediction of paravalvular AR than 2D TOE [68, 69].

Annulus measurements using 3D imaging with TOE, multislice CT and MRI have been studied and have been compared to 2D echocardiography. CT- and TOE-based annular measurements have been shown to both change valve sizing strategy and have a higher predictive value than 2D TOE for paravalvular AR [70–72]. When CT has been compared to 3D echocardiography, they were both found to predict paravalvular AR with equal accuracy [73]. Annulus diameters and areas for pre-procedural TAVI assessment by 3D-TOE are significantly smaller than multislice computer tomography (MSCT) with exception of sagittal diameters. Using sagittal diameters, both modalities predicted well final prosthesis size and excellent procedural results were obtained. 3D-TOE can thus be a useful alternative in patients with contraindications to MSCT [74].

On average, compared with CT, even 3D echo provides an annular area of just under 0.5 cm^2^, which is significantly closer than 2D echo but still allows for a potential discrepancy. This difference must be borne in mind when valve sizing using 3D echo which is sometimes required when CT is not possible. By and large however, CT is the preferred method for making this assessment.

(c) Root anatomy

Echocardiography also provides assessment of the relationship of the coronary arteries to the annulus and valve leaflets. Valve deployment should not compromise the coronary ostia, either from the device itself or from cusp calcification that may be shifted into the coronary arteries. The height of the coronary ostia from the base of the aortic valve leaflets ideally should be greater than 10 mm to prevent coronary arterial occlusion on implantation of the prosthesis [75, 76]. The relationship between the sinus capacity and valvular calcium should always be carefully evaluated.

(d) Significant left ventricular upper septal hypertrophy has traditionally been considered a relative contraindication to TAVI because of the risk of maldeployment [77–79]. Severe left ventricular hypertrophy with apical obliteration may preclude transapical TAVI, whilst marked angulation of the ascending aorta or aortic arch may be more suited to transapical delivery of the prosthesis [80]. With modern implantation techniques and valve choices, this is rarely a problem. It is nonetheless a noteworthy feature and may influence the choice of valve. TOE is also able to observe bulky and friable complex atheromatous plaques in the ascending aorta. These may impede the passage of the delivery system or result in complications. Measurements of the aortic sinuses, sinotubular junction and ascending and descending aorta on CT can also be made, and the extent of atherosclerotic plaque is likely associated with complications including stroke [81, 82].

**Fig. 1:**
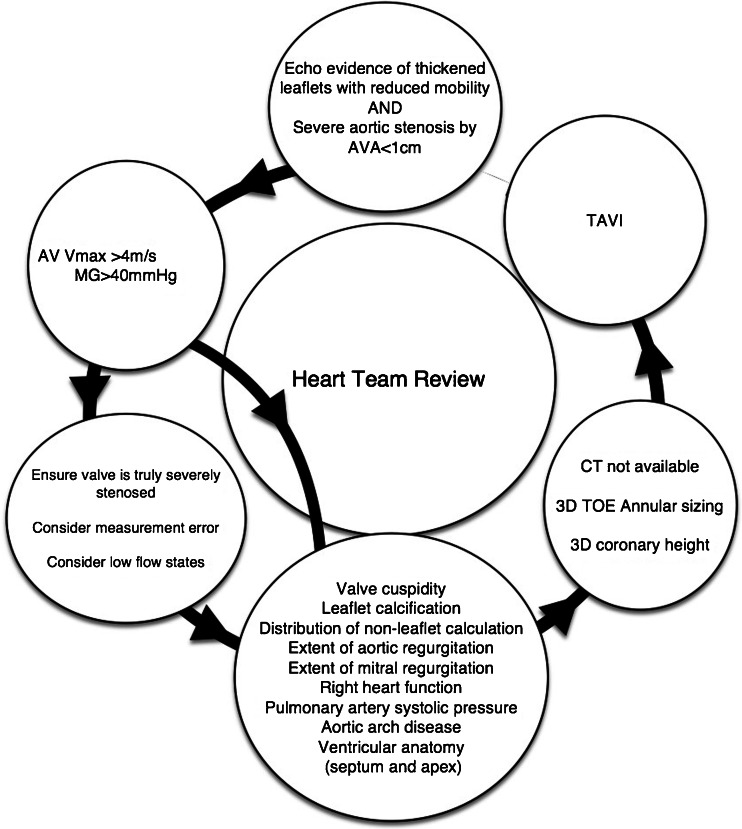
Flow diagram outlining the role of echocardiographic assessment of aortic stenosis in the workup prior to TAVI

A flow diagram describing the echocardiographic assessment of aortic stenosis prior to TAVI is detailed in Fig. 1.

## Conclusions

The development of transcatheter aortic valve implantation has been a seismic change in the management of aortic stenosis as an alternative intervention to surgical aortic valve replacement in appropriately selected patients. Selection of patients prior to TAVI requires optimal imaging, and although 2D TTE is the initial modality of choice, there may be cases where TOE where may required. Confirming the severity of aortic stenosis and the presence of concomitant factors such as mitral regurgitation and pulmonary hypertension is usually performed by TTE. However, the aortic valve is a complex structure and 3D echocardiography and CT are the preferred imaging modalities for assessing the anatomy and annular dimensions prior to TAVI and have been shown to change valve sizing strategy compared with 2D echo.
